# Tris(1,10-phenanthroline-κ^2^ *N*,*N*′)iron(II) μ-oxido-bis[trichloridoferrate(III)] ethanol hemisolvate

**DOI:** 10.1107/S1600536808035897

**Published:** 2008-11-08

**Authors:** Zhan-Xian Li, Ming-Ming Yu, Yu-Na Zhang, Liu-He Wei

**Affiliations:** a Department of Chemistry, Zhengzhou University, Zhengzhou 450001, People’s Republic of China

## Abstract

The title compound, [Fe(C_12_H_8_N_2_)_3_][Fe_2_Cl_6_O]·0.5CH_3_CH_2_OH, consists of one [Fe(phen)_3_]^2+^ cation (phen = 1,10-phen­anthroline), one [Fe_2_Cl_6_O]^2−^ anion and one half-mol­ecule of ethanol. In the cation, the Fe^II^ atom is coordinated by six N atoms from three phen ligands in a distorted octa­hedral geometry. In the bent anion, two Fe^III^ atoms are connected by a bridging oxide O atom [bridging angle = 160.6 (4)°], and each Fe^III^ atom is also coordinated by three Cl atoms, completing a distorted tetra­hedral geometry.

## Related literature

For general background, see: Hwang & Ha (2006 ▶); Potočňák *et al.* (2002 ▶); Zhou & Guo (2007 ▶). For a related structure, see: Aparici Plaza *et al.* (2007 ▶).
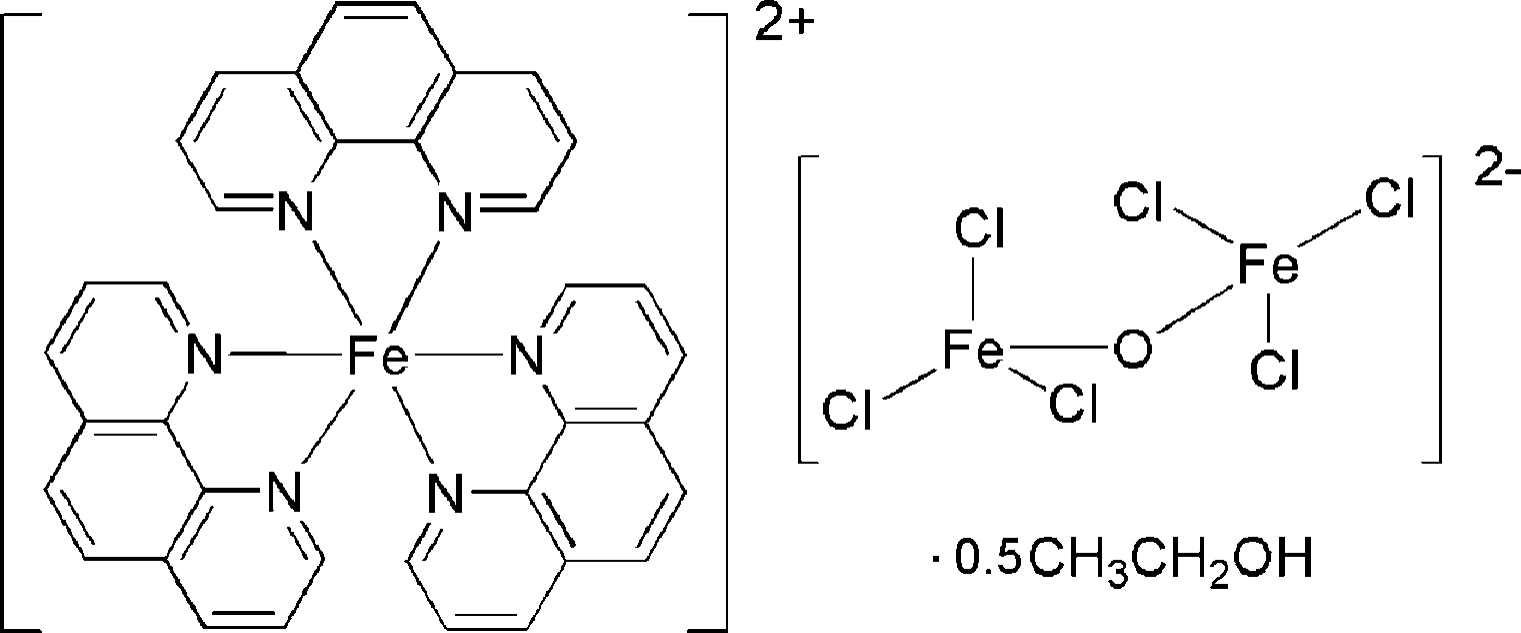


## Experimental

### 

#### Crystal data

[Fe(C_12_H_8_N_2_)_3_][Fe_2_Cl_6_O]·0.5C_2_H_6_O
*M* *_r_* = 959.89
Monoclinic, 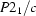

*a* = 15.3536 (18) Å
*b* = 13.1857 (16) Å
*c* = 20.897 (2) Å
β = 94.701 (2)°
*V* = 4216.4 (8) Å^3^
*Z* = 4
Mo *K*α radiation
μ = 1.44 mm^−1^
*T* = 294 (2) K
0.55 × 0.47 × 0.42 mm

#### Data collection

Bruker SMART 1K CCD area-detector diffractometer
Absorption correction: multi-scan (*SADABS*; Sheldrick, 1996 ▶) *T*_min_ = 0.452, *T*_max_ = 0.544
20409 measured reflections
7434 independent reflections
4248 reflections with *I* > 2σ(*I*)
*R*_int_ = 0.058

#### Refinement

*R*[*F* ^2^ > 2σ(*F* ^2^)] = 0.064
*wR*(*F* ^2^) = 0.212
*S* = 1.02
7434 reflections
498 parameters
3 restraints
H-atom parameters constrained
Δρ_max_ = 0.84 e Å^−3^
Δρ_min_ = −0.39 e Å^−3^

### 

Data collection: *SMART* (Bruker, 2007 ▶); cell refinement: *SAINT* (Bruker, 2007 ▶); data reduction: *SAINT*; program(s) used to solve structure: *SHELXTL* (Sheldrick, 2008 ▶); program(s) used to refine structure: *SHELXTL*; molecular graphics: *SHELXTL*; software used to prepare material for publication: *SHELXTL*.

## Supplementary Material

Crystal structure: contains datablocks I, global. DOI: 10.1107/S1600536808035897/hy2160sup1.cif

Structure factors: contains datablocks I. DOI: 10.1107/S1600536808035897/hy2160Isup2.hkl

Additional supplementary materials:  crystallographic information; 3D view; checkCIF report

## Figures and Tables

**Table 1:** Selected bond lengths (Å)

Fe1—N4	1.958 (6)
Fe1—N2	1.970 (5)
Fe1—N6	1.979 (5)
Fe1—N1	1.980 (5)
Fe1—N3	1.982 (5)
Fe1—N5	1.985 (5)
Fe2—O1	1.735 (5)
Fe2—Cl4	2.204 (3)
Fe2—Cl6	2.223 (2)
Fe2—Cl5	2.231 (2)
Fe3—O1	1.756 (5)
Fe3—Cl2	2.213 (2)
Fe3—Cl3	2.227 (2)
Fe3—Cl1	2.227 (2)

**Fig. 1.**
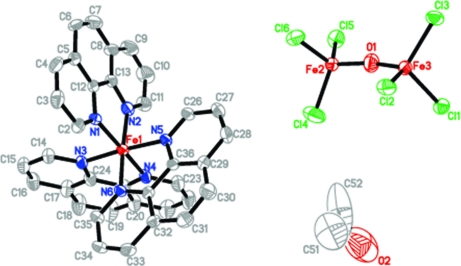
The structures of the cation, anion and solvate molecule in the title compoud. Displacement ellipsoids are drawn at the 30% probability level.

## supplementary crystallographic information

### Comment

1,10-Phenanthroline (phen) is a widely utilized chelating ligand in coordination chemistry and a lot of complexes with phen as a ligand have been reported (Hwang & Ha, 2006; Potocnak *et al.*, 2002; Zhou & Guo, 2007). Plaza *et al.* (2007) has reported the structure of a complex with [Fe(phen)_3_]^2+^ as cation and simple halide as counter-ion. We report here the synthesis and crystal structure of the title compound, in which an oxide-bridged iron(III) complex acts as counter-ion.

The title compound comprises one [Fe(phen)_3_]^2+^ cation, one [Fe_2_Cl_6_O]^2-^ anion and a half of solvent ethanol molecule (Fig. 1). The Fe^II^ atom in the cation is coordinated by six N atoms from three phen ligands, forming a distorted FeN_6_ octahedral geometry. The Fe—N bond lengths are in the normal range (Table 1). In the anion, two Fe^III^ atoms are bridged by an oxide O atom and is also coordinated by three Cl atoms in a distorted tetrahedral geometry. The Fe—Cl bond distances are in the range of 2.204 (3) to 2.231 (2) Å. The distance betweeen the Fe^III^ atoms (Fe2 and Fe3) bridged by the O1 atom is 3.441 (5) Å [Fe2—O1 = 1.735 (5) and Fe3—O1 = 1.756 (5) Å].

### Experimental

Fe(ClO_4_)_2_.6H_2_O (0.036 g, 0.1 mmol) and 1,10-phenanthroline (0.054 g, 0.3 mmol) are dissolved in 15 ml of water and ethanol (1:2 in volume), resulting in a pale green solution, which was transfered to the left hand of an H-shaped tube. An aqueous solution (15 ml) of FeCl_3_ (0.032 g, 0.2 mmol) was placed in the right hand of the tube. An ethanol solution serving as the diffusion solvent was put in the connecting part between the left and right hands of the tube. The reaction container was kept at room temperature. Dark brown crystals suitable for X-ray diffraction analysis were obtained in the connecting part of the H-shaped tube after two months (yield 60% based on Fe).

### Refinement

H atoms were positioned geometrically and refined as riding atoms, with C—H = 0.93 (aromatic), 0.97 (CH_2_) and 0.96 (CH_3_) Å and  *U*_iso_(H) = 1.2(1.5 for methyl)*U*_eq_(C), and with O—H = 0.82 Å and *U*_iso_(H) = 1.5*U*_eq_(O).

### Crystal data

**Table:**

[Fe(C_12_H_8_N_2_)_3_][Fe_2_Cl_6_O]·0.5C_2_H_6_O	*F*_000_ = 1932
*M**_r_* = 959.89	*D*_x_ = 1.512 Mg m^−^^3^
Monoclinic, *P*2_1_/*c*	Mo *K*α radiation λ = 0.71073 Å
Hall symbol: -P 2ybc	Cell parameters from 20409 reflections
*a* = 15.3536 (18) Å	θ = 1.8–25.0º
*b* = 13.1857 (16) Å	µ = 1.44 mm^−^^1^
*c* = 20.897 (2) Å	*T* = 294 (2) K
β = 94.701 (2)º	Block, brown
*V* = 4216.4 (8) Å^3^	0.55 × 0.47 × 0.42 mm
*Z* = 4	

### Data collection

**Table:**

Bruker SMART 1K CCD area-detector diffractometer	7434 independent reflections
Radiation source: fine-focus sealed tube	4248 reflections with *I* > 2σ(*I*)
Monochromator: graphite	*R*_int_ = 0.058
*T* = 294(2) K	θ_max_ = 25.0º
φ and ω scans	θ_min_ = 1.8º
Absorption correction: multi-scan(SADABS; Sheldrick, 1996)	*h* = −18→14
*T*_min_ = 0.452, *T*_max_ = 0.544	*k* = −15→15
20409 measured reflections	*l* = −17→24

### Refinement

**Table:**

Refinement on *F*^2^	Secondary atom site location: difference Fourier map
Least-squares matrix: full	Hydrogen site location: inferred from neighbouring sites
*R*[*F*^2^ > 2σ(*F*^2^)] = 0.064	H-atom parameters constrained
*wR*(*F*^2^) = 0.212	*w* = 1/[σ^2^(*F*_o_^2^) + (0.1185*P*)^2^] where *P* = (*F*_o_^2^ + 2*F*_c_^2^)/3
*S* = 1.03	(Δ/σ)_max_ = 0.018
7434 reflections	Δρ_max_ = 0.84 e Å^−^^3^
498 parameters	Δρ_min_ = −0.39 e Å^−^^3^
3 restraints	Extinction correction: none
Primary atom site location: structure-invariant direct methods	

### Fractional atomic coordinates and isotropic or equivalent isotropic displacement parameters (Å^2^)

**Table:**

	*x*	*y*	*z*	*U*_iso_*/*U*_eq_	Occ. (<1)
Fe1	0.25729 (6)	0.78424 (6)	0.06260 (4)	0.0388 (3)	
Fe2	0.21293 (7)	0.12200 (8)	0.71711 (5)	0.0533 (3)	
Fe3	0.34590 (7)	0.14054 (7)	0.85736 (5)	0.0505 (3)	
Cl1	0.27250 (13)	0.18682 (15)	0.94022 (9)	0.0645 (5)	
Cl2	0.43389 (15)	0.26757 (15)	0.83747 (11)	0.0765 (6)	
Cl3	0.42517 (14)	0.00431 (14)	0.88656 (10)	0.0696 (6)	
Cl4	0.1307 (2)	0.2590 (2)	0.70798 (16)	0.1203 (11)	
Cl5	0.12833 (15)	−0.01536 (17)	0.70469 (11)	0.0815 (7)	
Cl6	0.30925 (16)	0.12534 (15)	0.64332 (11)	0.0811 (7)	
O1	0.2707 (4)	0.1130 (5)	0.7918 (3)	0.0874 (18)	
N1	0.3671 (4)	0.7608 (4)	0.0213 (2)	0.0440 (13)	
N2	0.2292 (4)	0.6451 (4)	0.0334 (3)	0.0500 (15)	
N3	0.1978 (3)	0.8384 (4)	−0.0180 (2)	0.0431 (13)	
N4	0.1411 (4)	0.7998 (4)	0.0936 (3)	0.0467 (14)	
N5	0.3138 (3)	0.7370 (4)	0.1461 (2)	0.0393 (12)	
N6	0.2932 (3)	0.9184 (4)	0.0981 (2)	0.0400 (12)	
C2	0.4338 (4)	0.8226 (5)	0.0168 (3)	0.0475 (17)	
H27	0.4333	0.8864	0.0357	0.057*	
C3	0.5060 (5)	0.7928 (7)	−0.0164 (3)	0.063 (2)	
H36	0.5519	0.8378	−0.0205	0.076*	
C4	0.5088 (6)	0.6978 (7)	−0.0427 (3)	0.068 (2)	
H33	0.5571	0.6775	−0.0636	0.082*	
C5	0.4394 (6)	0.6325 (6)	−0.0380 (3)	0.057 (2)	
C6	0.4341 (7)	0.5289 (7)	−0.0641 (3)	0.076 (3)	
H38	0.4798	0.5029	−0.0856	0.091*	
C7	0.3610 (8)	0.4702 (7)	−0.0565 (4)	0.085 (3)	
H47	0.3588	0.4049	−0.0734	0.102*	
C8	0.2901 (6)	0.5047 (6)	−0.0244 (4)	0.064 (2)	
C9	0.2164 (8)	0.4478 (6)	−0.0139 (4)	0.085 (3)	
H32	0.2108	0.3821	−0.0300	0.102*	
C10	0.1525 (7)	0.4877 (7)	0.0196 (5)	0.088 (3)	
H49	0.1036	0.4496	0.0277	0.105*	
C11	0.1617 (5)	0.5860 (6)	0.0415 (3)	0.062 (2)	
H37	0.1169	0.6127	0.0637	0.075*	
C12	0.3679 (5)	0.6666 (5)	−0.0059 (3)	0.0440 (16)	
C13	0.2948 (5)	0.6026 (5)	0.0009 (3)	0.0490 (17)	
C14	0.2293 (5)	0.8619 (5)	−0.0731 (3)	0.0508 (18)	
H26	0.2890	0.8547	−0.0765	0.061*	
C15	0.1775 (5)	0.8967 (6)	−0.1261 (4)	0.063 (2)	
H45	0.2029	0.9139	−0.1635	0.076*	
C16	0.0899 (6)	0.9056 (6)	−0.1234 (4)	0.069 (2)	
H20	0.0548	0.9260	−0.1594	0.083*	
C17	0.0521 (5)	0.8837 (6)	−0.0653 (4)	0.066 (2)	
C18	−0.0379 (6)	0.8918 (8)	−0.0552 (4)	0.087 (3)	
H44	−0.0775	0.9122	−0.0889	0.105*	
C19	−0.0667 (5)	0.8707 (8)	0.0018 (4)	0.092 (3)	
H50	−0.1263	0.8757	0.0067	0.111*	
C20	−0.0097 (5)	0.8408 (7)	0.0554 (4)	0.068 (2)	
C21	−0.0344 (5)	0.8189 (7)	0.1172 (5)	0.082 (3)	
H46	−0.0928	0.8239	0.1257	0.099*	
C22	0.0263 (6)	0.7905 (7)	0.1645 (4)	0.076 (2)	
H35	0.0095	0.7769	0.2054	0.092*	
C23	0.1141 (5)	0.7815 (6)	0.1520 (4)	0.062 (2)	
H43	0.1549	0.7622	0.1851	0.075*	
C24	0.1109 (4)	0.8498 (5)	−0.0134 (3)	0.0482 (17)	
C25	0.0799 (4)	0.8296 (5)	0.0472 (3)	0.0485 (17)	
C26	0.3254 (4)	0.6437 (5)	0.1694 (3)	0.0484 (17)	
H28	0.3050	0.5893	0.1442	0.058*	
C27	0.3662 (5)	0.6239 (5)	0.2291 (4)	0.0580 (19)	
H18	0.3717	0.5573	0.2434	0.070*	
C28	0.3980 (5)	0.6995 (6)	0.2667 (3)	0.060 (2)	
H2	0.4250	0.6858	0.3072	0.072*	
C29	0.3903 (5)	0.7982 (5)	0.2448 (3)	0.0528 (18)	
C30	0.4249 (6)	0.8884 (6)	0.2784 (4)	0.076 (3)	
H42	0.4580	0.8807	0.3174	0.091*	
C31	0.4105 (6)	0.9821 (6)	0.2548 (4)	0.076 (3)	
H48	0.4306	1.0379	0.2789	0.092*	
C32	0.3649 (5)	0.9978 (5)	0.1934 (3)	0.0542 (19)	
C33	0.3478 (5)	1.0945 (5)	0.1650 (4)	0.062 (2)	
H34	0.3652	1.1535	0.1868	0.075*	
C34	0.3054 (5)	1.0992 (5)	0.1050 (4)	0.058 (2)	
H25	0.2939	1.1618	0.0857	0.070*	
C35	0.2796 (4)	1.0107 (5)	0.0728 (3)	0.0489 (17)	
H19	0.2515	1.0159	0.0318	0.059*	
C36	0.3465 (4)	0.8139 (4)	0.1835 (3)	0.0396 (15)	
C37	0.3342 (4)	0.9128 (5)	0.1575 (3)	0.0405 (15)	
C51	0.2017 (13)	0.6513 (18)	0.8088 (10)	0.128 (9)	0.50
H51A	0.1715	0.7143	0.8008	0.191*	0.50
H51B	0.2320	0.6333	0.7721	0.191*	0.50
H51C	0.2429	0.6583	0.8456	0.191*	0.50
C52	0.1376 (18)	0.570 (2)	0.8211 (13)	0.29 (4)	0.50
H52A	0.1042	0.5525	0.7813	0.347*	0.50
H52B	0.1682	0.5104	0.8376	0.347*	0.50
O2	0.0796 (14)	0.6063 (17)	0.8669 (11)	0.183 (10)	0.50
H2A	0.0895	0.6664	0.8746	0.275*	0.50

### Atomic displacement parameters (Å^2^)

**Table:**

	*U*^11^	*U*^22^	*U*^33^	*U*^12^	*U*^13^	*U*^23^
Fe1	0.0416 (5)	0.0331 (5)	0.0421 (5)	0.0006 (4)	0.0060 (4)	0.0023 (4)
Fe2	0.0609 (7)	0.0507 (6)	0.0489 (6)	0.0022 (5)	0.0082 (5)	0.0018 (5)
Fe3	0.0615 (7)	0.0439 (6)	0.0467 (6)	−0.0040 (5)	0.0082 (5)	0.0014 (4)
Cl1	0.0630 (13)	0.0672 (12)	0.0658 (12)	−0.0007 (10)	0.0218 (10)	0.0013 (9)
Cl2	0.0889 (16)	0.0510 (11)	0.0956 (16)	−0.0046 (10)	0.0435 (13)	0.0125 (10)
Cl3	0.0819 (14)	0.0420 (10)	0.0844 (14)	0.0027 (9)	0.0029 (11)	−0.0028 (9)
Cl4	0.116 (2)	0.0853 (18)	0.166 (3)	0.0400 (16)	0.050 (2)	0.0337 (17)
Cl5	0.0851 (16)	0.0794 (15)	0.0809 (15)	−0.0258 (12)	0.0116 (12)	−0.0001 (11)
Cl6	0.1018 (17)	0.0544 (12)	0.0941 (16)	−0.0114 (11)	0.0504 (13)	−0.0036 (11)
O1	0.106 (5)	0.089 (4)	0.064 (4)	−0.003 (3)	−0.006 (3)	−0.002 (3)
N1	0.051 (4)	0.039 (3)	0.043 (3)	0.012 (3)	0.010 (3)	0.006 (2)
N2	0.062 (4)	0.042 (3)	0.045 (3)	−0.007 (3)	−0.003 (3)	0.004 (3)
N3	0.045 (3)	0.042 (3)	0.044 (3)	−0.006 (2)	0.009 (3)	0.007 (2)
N4	0.048 (3)	0.044 (3)	0.049 (3)	−0.004 (3)	0.008 (3)	0.012 (3)
N5	0.045 (3)	0.031 (3)	0.043 (3)	−0.002 (2)	0.007 (2)	0.004 (2)
N6	0.043 (3)	0.030 (3)	0.049 (3)	0.001 (2)	0.012 (3)	0.003 (2)
C2	0.040 (4)	0.054 (4)	0.051 (4)	−0.002 (3)	0.015 (3)	0.002 (3)
C3	0.051 (5)	0.087 (6)	0.052 (5)	0.000 (4)	0.009 (4)	0.017 (4)
C4	0.069 (6)	0.097 (7)	0.041 (4)	0.037 (5)	0.014 (4)	0.004 (4)
C5	0.075 (6)	0.062 (5)	0.036 (4)	0.026 (4)	0.006 (4)	0.010 (3)
C6	0.116 (8)	0.074 (6)	0.038 (4)	0.056 (6)	0.014 (5)	−0.006 (4)
C7	0.130 (9)	0.054 (5)	0.067 (6)	0.026 (6)	−0.008 (6)	−0.008 (4)
C8	0.097 (6)	0.043 (4)	0.051 (5)	0.016 (4)	0.005 (4)	−0.011 (3)
C9	0.131 (9)	0.038 (5)	0.082 (7)	−0.009 (5)	−0.015 (6)	−0.006 (4)
C10	0.107 (8)	0.054 (5)	0.101 (8)	−0.024 (5)	0.003 (6)	−0.003 (5)
C11	0.073 (5)	0.049 (4)	0.064 (5)	−0.016 (4)	0.005 (4)	0.001 (4)
C12	0.056 (4)	0.044 (4)	0.033 (4)	0.016 (3)	0.006 (3)	0.005 (3)
C13	0.066 (5)	0.043 (4)	0.038 (4)	0.006 (3)	0.000 (3)	0.005 (3)
C14	0.052 (4)	0.058 (4)	0.043 (4)	−0.003 (3)	0.002 (3)	0.006 (3)
C15	0.068 (6)	0.072 (5)	0.052 (5)	−0.004 (4)	0.014 (4)	0.013 (4)
C16	0.073 (6)	0.087 (6)	0.045 (5)	−0.001 (5)	−0.010 (4)	0.015 (4)
C17	0.055 (5)	0.088 (6)	0.053 (5)	−0.005 (4)	0.002 (4)	0.009 (4)
C18	0.053 (5)	0.141 (9)	0.066 (6)	0.002 (5)	−0.010 (4)	0.025 (6)
C19	0.039 (5)	0.160 (10)	0.078 (7)	0.008 (5)	0.003 (4)	0.023 (6)
C20	0.041 (5)	0.097 (6)	0.068 (6)	0.005 (4)	0.009 (4)	0.019 (4)
C21	0.044 (5)	0.116 (8)	0.089 (7)	0.008 (5)	0.020 (5)	0.013 (6)
C22	0.060 (6)	0.103 (7)	0.070 (6)	−0.001 (5)	0.025 (5)	0.020 (5)
C23	0.065 (5)	0.066 (5)	0.056 (5)	0.001 (4)	0.008 (4)	0.014 (4)
C24	0.042 (4)	0.049 (4)	0.053 (4)	−0.008 (3)	0.000 (3)	0.006 (3)
C25	0.042 (4)	0.056 (4)	0.047 (4)	−0.005 (3)	0.005 (3)	0.007 (3)
C26	0.057 (4)	0.034 (4)	0.055 (4)	0.001 (3)	0.010 (4)	0.004 (3)
C27	0.069 (5)	0.046 (4)	0.059 (5)	−0.001 (4)	0.004 (4)	0.010 (4)
C28	0.072 (5)	0.062 (5)	0.046 (4)	0.001 (4)	0.003 (4)	0.019 (4)
C29	0.063 (5)	0.054 (4)	0.041 (4)	0.004 (4)	0.007 (3)	−0.003 (3)
C30	0.104 (7)	0.069 (6)	0.051 (5)	0.008 (5)	−0.019 (5)	−0.005 (4)
C31	0.125 (8)	0.053 (5)	0.048 (5)	−0.003 (5)	−0.010 (5)	−0.019 (4)
C32	0.079 (5)	0.038 (4)	0.048 (4)	0.000 (4)	0.020 (4)	−0.005 (3)
C33	0.085 (6)	0.037 (4)	0.067 (5)	−0.005 (4)	0.018 (4)	−0.009 (3)
C34	0.076 (5)	0.031 (4)	0.068 (5)	0.010 (3)	0.009 (4)	0.003 (3)
C35	0.058 (4)	0.035 (4)	0.054 (4)	0.005 (3)	0.008 (3)	0.006 (3)
C36	0.045 (4)	0.034 (3)	0.041 (4)	0.000 (3)	0.011 (3)	0.001 (3)
C37	0.046 (4)	0.040 (4)	0.036 (4)	0.001 (3)	0.006 (3)	−0.002 (3)
C51	0.12 (2)	0.16 (2)	0.105 (18)	0.066 (18)	0.018 (15)	−0.036 (16)
C52	0.12 (3)	0.45 (8)	0.28 (5)	−0.07 (4)	−0.10 (3)	0.30 (6)
O2	0.18 (2)	0.147 (18)	0.21 (2)	0.007 (16)	−0.078 (18)	−0.017 (16)

### Geometric parameters (Å, °)

**Table:**

Fe1—N4	1.958 (6)	C14—H26	0.9300
Fe1—N2	1.970 (5)	C15—C16	1.356 (10)
Fe1—N6	1.979 (5)	C15—H45	0.9300
Fe1—N1	1.980 (5)	C16—C17	1.418 (10)
Fe1—N3	1.982 (5)	C16—H20	0.9300
Fe1—N5	1.985 (5)	C17—C18	1.419 (11)
Fe2—O1	1.735 (5)	C17—C24	1.425 (9)
Fe2—Cl4	2.204 (3)	C18—C19	1.335 (11)
Fe2—Cl6	2.223 (2)	C18—H44	0.9300
Fe2—Cl5	2.231 (2)	C19—C20	1.419 (11)
Fe3—O1	1.756 (5)	C19—H50	0.9300
Fe3—Cl2	2.213 (2)	C20—C21	1.405 (11)
Fe3—Cl3	2.227 (2)	C20—C25	1.408 (10)
Fe3—Cl1	2.227 (2)	C21—C22	1.355 (11)
N1—C2	1.318 (8)	C21—H46	0.9300
N1—C12	1.367 (8)	C22—C23	1.399 (11)
N2—C11	1.319 (9)	C22—H35	0.9300
N2—C13	1.379 (9)	C23—H43	0.9300
N3—C14	1.323 (8)	C24—C25	1.415 (9)
N3—C24	1.353 (8)	C26—C27	1.373 (9)
N4—C23	1.343 (8)	C26—H28	0.9300
N4—C25	1.353 (8)	C27—C28	1.337 (10)
N5—C26	1.330 (7)	C27—H18	0.9300
N5—C36	1.351 (8)	C28—C29	1.381 (9)
N6—C35	1.337 (7)	C28—H2	0.9300
N6—C37	1.346 (7)	C29—C36	1.412 (9)
C2—C3	1.411 (10)	C29—C30	1.458 (10)
C2—H27	0.9300	C30—C31	1.342 (11)
C3—C4	1.370 (11)	C30—H42	0.9300
C3—H36	0.9300	C31—C32	1.425 (10)
C4—C5	1.380 (11)	C31—H48	0.9300
C4—H33	0.9300	C32—C37	1.408 (9)
C5—C12	1.406 (10)	C32—C33	1.421 (9)
C5—C6	1.471 (11)	C33—C34	1.365 (10)
C6—C7	1.383 (13)	C33—H34	0.9300
C6—H38	0.9300	C34—C35	1.389 (9)
C7—C8	1.399 (12)	C34—H25	0.9300
C7—H47	0.9300	C35—H19	0.9300
C8—C9	1.391 (12)	C36—C37	1.420 (8)
C8—C13	1.395 (10)	C51—C52	1.487 (18)
C9—C10	1.358 (13)	C51—H51A	0.9600
C9—H32	0.9300	C51—H51B	0.9600
C10—C11	1.377 (11)	C51—H51C	0.9600
C10—H49	0.9300	C52—O2	1.440 (19)
C11—H37	0.9300	C52—H52A	0.9700
C12—C13	1.420 (10)	C52—H52B	0.9700
C14—C15	1.387 (9)	O2—H2A	0.8200
			
N4—Fe1—N2	91.0 (2)	C15—C14—H26	118.4
N4—Fe1—N6	90.9 (2)	C16—C15—C14	120.0 (7)
N2—Fe1—N6	174.7 (2)	C16—C15—H45	120.0
N4—Fe1—N1	172.7 (2)	C14—C15—H45	120.0
N2—Fe1—N1	83.8 (2)	C15—C16—C17	119.8 (7)
N6—Fe1—N1	94.7 (2)	C15—C16—H20	120.1
N4—Fe1—N3	82.8 (2)	C17—C16—H20	120.1
N2—Fe1—N3	89.9 (2)	C18—C17—C16	125.7 (7)
N6—Fe1—N3	95.3 (2)	C18—C17—C24	118.4 (7)
N1—Fe1—N3	92.1 (2)	C16—C17—C24	115.8 (7)
N4—Fe1—N5	95.0 (2)	C19—C18—C17	121.0 (7)
N2—Fe1—N5	92.8 (2)	C19—C18—H44	119.5
N6—Fe1—N5	82.1 (2)	C17—C18—H44	119.5
N1—Fe1—N5	90.4 (2)	C18—C19—C20	122.3 (8)
N3—Fe1—N5	176.5 (2)	C18—C19—H50	118.8
O1—Fe2—Cl4	112.4 (2)	C20—C19—H50	118.8
O1—Fe2—Cl6	107.8 (2)	C21—C20—C25	115.5 (7)
Cl4—Fe2—Cl6	109.44 (11)	C21—C20—C19	125.9 (7)
O1—Fe2—Cl5	107.4 (2)	C25—C20—C19	118.6 (7)
Cl4—Fe2—Cl5	109.35 (12)	C22—C21—C20	120.4 (7)
Cl6—Fe2—Cl5	110.45 (9)	C22—C21—H46	119.8
O1—Fe3—Cl2	112.5 (2)	C20—C21—H46	119.8
O1—Fe3—Cl3	110.9 (2)	C21—C22—C23	120.2 (8)
Cl2—Fe3—Cl3	109.50 (9)	C21—C22—H35	119.9
O1—Fe3—Cl1	108.7 (2)	C23—C22—H35	119.9
Cl2—Fe3—Cl1	107.12 (9)	N4—C23—C22	121.9 (7)
Cl3—Fe3—Cl1	107.94 (9)	N4—C23—H43	119.0
Fe2—O1—Fe3	160.6 (4)	C22—C23—H43	119.0
C2—N1—C12	119.9 (6)	N3—C24—C25	116.6 (6)
C2—N1—Fe1	129.0 (5)	N3—C24—C17	123.3 (7)
C12—N1—Fe1	111.1 (4)	C25—C24—C17	120.0 (6)
C11—N2—C13	115.8 (6)	N4—C25—C20	124.9 (7)
C11—N2—Fe1	131.7 (6)	N4—C25—C24	115.5 (6)
C13—N2—Fe1	112.4 (5)	C20—C25—C24	119.6 (6)
C14—N3—C24	117.9 (6)	N5—C26—C27	123.1 (6)
C14—N3—Fe1	130.4 (5)	N5—C26—H28	118.4
C24—N3—Fe1	111.7 (4)	C27—C26—H28	118.4
C23—N4—C25	117.1 (6)	C28—C27—C26	120.6 (7)
C23—N4—Fe1	129.8 (5)	C28—C27—H18	119.7
C25—N4—Fe1	113.1 (4)	C26—C27—H18	119.7
C26—N5—C36	116.7 (5)	C27—C28—C29	119.4 (7)
C26—N5—Fe1	130.4 (4)	C27—C28—H2	120.3
C36—N5—Fe1	112.8 (4)	C29—C28—H2	120.3
C35—N6—C37	117.4 (5)	C28—C29—C36	117.3 (6)
C35—N6—Fe1	129.5 (4)	C28—C29—C30	126.3 (7)
C37—N6—Fe1	113.1 (4)	C36—C29—C30	116.4 (6)
N1—C2—C3	120.7 (7)	C31—C30—C29	122.1 (7)
N1—C2—H27	119.7	C31—C30—H42	118.9
C3—C2—H27	119.7	C29—C30—H42	118.9
C4—C3—C2	120.2 (8)	C30—C31—C32	121.2 (7)
C4—C3—H36	119.9	C30—C31—H48	119.4
C2—C3—H36	119.9	C32—C31—H48	119.4
C3—C4—C5	119.5 (7)	C37—C32—C33	116.6 (6)
C3—C4—H33	120.2	C37—C32—C31	118.8 (6)
C5—C4—H33	120.3	C33—C32—C31	124.5 (7)
C4—C5—C12	118.2 (7)	C34—C33—C32	118.8 (6)
C4—C5—C6	124.9 (8)	C34—C33—H34	120.6
C12—C5—C6	116.9 (8)	C32—C33—H34	120.6
C7—C6—C5	119.9 (8)	C33—C34—C35	120.2 (6)
C7—C6—H38	120.1	C33—C34—H25	119.9
C5—C6—H38	120.1	C35—C34—H25	119.9
C6—C7—C8	122.9 (8)	N6—C35—C34	123.0 (6)
C6—C7—H47	118.6	N6—C35—H19	118.5
C8—C7—H47	118.6	C34—C35—H19	118.5
C9—C8—C13	117.2 (8)	N5—C36—C29	122.8 (6)
C9—C8—C7	125.1 (8)	N5—C36—C37	115.9 (5)
C13—C8—C7	117.7 (9)	C29—C36—C37	121.3 (6)
C10—C9—C8	120.2 (8)	N6—C37—C32	124.0 (6)
C10—C9—H32	119.9	N6—C37—C36	116.1 (5)
C8—C9—H32	119.9	C32—C37—C36	119.9 (6)
C9—C10—C11	118.5 (9)	C52—C51—H51A	109.3
C9—C10—H49	120.7	C52—C51—H51B	109.6
C11—C10—H49	120.7	H51A—C51—H51B	109.5
N2—C11—C10	125.0 (9)	C52—C51—H51C	109.5
N2—C11—H37	117.5	H51A—C51—H51C	109.5
C10—C11—H37	117.5	H51B—C51—H51C	109.5
N1—C12—C5	121.5 (7)	O2—C52—C51	109.5 (17)
N1—C12—C13	117.7 (6)	O2—C52—H52A	109.9
C5—C12—C13	120.8 (7)	C51—C52—H52A	109.7
N2—C13—C8	123.1 (7)	O2—C52—H52B	109.8
N2—C13—C12	115.0 (6)	C51—C52—H52B	109.7
C8—C13—C12	121.9 (7)	H52A—C52—H52B	108.2
N3—C14—C15	123.2 (7)	C52—O2—H2A	109.5
N3—C14—H26	118.4		
